# OrgConv: detection of gene conversion using consensus sequences and its application in plant mitochondrial and chloroplast homologs

**DOI:** 10.1186/1471-2105-11-114

**Published:** 2010-03-02

**Authors:** Weilong Hao

**Affiliations:** 1 Department of Biology, Indiana University, Bloomington, IN 47405, USA

## Abstract

**Background:**

The ancestry of mitochondria and chloroplasts traces back to separate endosymbioses of once free-living bacteria. The highly reduced genomes of these two organelles therefore contain very distant homologs that only recently have been shown to recombine inside the mitochondrial genome. Detection of gene conversion between mitochondrial and chloroplast homologs was previously impossible due to the lack of suitable computer programs. Recently, I developed a novel method and have, for the first time, discovered recurrent gene conversion between chloroplast mitochondrial genes. The method will further our understanding of plant organellar genome evolution and help identify and remove gene regions with incongruent phylogenetic signals for several genes widely used in plant systematics. Here, I implement such a method that is available in a user friendly web interface.

**Results:**

OrgConv (**Org**anellar **Conv**ersion) is a computer package developed for detection of gene conversion between mitochondrial and chloroplast homologous genes. OrgConv is available in two forms; source code can be installed and run on a Linux platform and a web interface is available on multiple operating systems. The input files of the feature program are two multiple sequence alignments from different organellar compartments in FASTA format. The program compares every examined sequence against the consensus sequence of each sequence alignment rather than exhaustively examining every possible combination. Making use of consensus sequences significantly reduces the number of comparisons and therefore reduces overall computational time, which allows for analysis of very large datasets. Most importantly, with the significantly reduced number of comparisons, the statistical power remains high in the face of correction for multiple tests.

**Conclusions:**

Both the source code and the web interface of OrgConv are available for free from the OrgConv website http://www.indiana.edu/~orgconv. Although OrgConv has been developed with main focus on detection of gene conversion between mitochondrial and chloroplast genes, it may also be used for detection of gene conversion between any two distinct groups of homologous sequences.

## Background

Efforts to detect gene conversion and homologous recombination (HR) have increased in the past two decades [1,2]. This has sparked the development of many computer programs, such as RDP[3], geneconv[4], Max*χ*^2^[5], Homoplasy test [6], Phi[7] and many others. In general, the vast majority of interspecific HR events involve closely related species [8-11], and the frequency of HR tends to decrease sharply with the level of relatedness between donor and recipient [12-14]. Nonetheless, several cases of gene conversion between distantly related homologous sequences have been reported in recent years [15-17]. Mitochondria (mt) and chloroplasts (cp) originated from endosymbiotic bacteria and last shared common ancestry some 2 billion years ago. Plant mitochondrial genomes harbor a significant amount of chloroplast sequences (up to 8.8% of the complete mitochondrial genomes) due to intracellular gene transfer from chloroplast to mitochondria [18,19]. The coexistence of homologous genes inside the mitochondrial genome creates the potential for gene conversion between ancient homologs. Plant mitochondrial and chloroplast genomes share 3 ribosomal RNA genes and about half of the 40 protein coding genes, which together serve as the substrate for recombination. The discovery of several chimeric plant mitochondrial genes, in this case between native and horizontally transferred mitochondrial genes [20,21], further suggest that mitochondrial genes are involved in recombination/conversion during or after DNA exchange events. Despite this abundance of factors that would seem to facilitate conversion in mitochondrial genes, evidence of gene conversion from ancient chloroplast homologs into mitochondrial genes has, until recently, not been shown. One possible reason is that the relatively low substitution rate in both plant mitochondrial and chloroplast genes [22,23] prevents mt-cp conversion from being detected, since both empirical and simulation studies have shown that all existing programs are not sensitive at very low sequence diversity [24-27]. In this article, I describe a new method [28] that makes use of consensus sequences, which have good computational efficiency and retain high statistical power. The development of the method led us to a discovery of recurrent conversion between the mitochondrial and chloroplast homologs of the alpha subunit of ATP synthase in the mitochondrial genes [28]. Here, I implement the method into a computer program, and make it available for the public in both source code and a user friendly web interface.

## Implementation

### The core calculation for conversion identification

The core calculation for detection of conversion in OrgConv was conducted using a method modified from the RDP (Recombination Detection Program) method [3]. The RDP method compares three sequences each time by only examining informative sites. The probability to observe one recombination follows a binomial distribution:

where *L *is the length of informative sites, *N *is the length of the putative recombinant segment, *M *is the number of common nucleotides shared between the putative recombinant sequences, and *p *is the proportion of nucleotides common between the same pair of sequences. There are  non-overlapping windows of size *N *in the sequence (*L *sites). The term  was used in the RDP method to correct for multiple windows.

In this study, two improvements were made to the above calculation. 1), the parameter *p *(the proportion of nucleotides common between sequences) was calculated from the sequence excluding the examined region instead of from the entire sequence. The calculation based on the entire sequence in the original RDP method is under the null hypothesis that there is no recombination. However, when there is recombination, the proportion of nucleotides common between the entire donor and recipient sequences is inflated because of the recombinant region, and consequently the calculated probability *P *will be less significant than it should be. It would therefore be more appropriate to exclude the examined region from the overall *p *calculation. 2), in addition to the term , a second term (*L - N*) was introduced to correct for multiple windows. In this study, calculation was performed in sliding-windows by incrementing one informative site at a time. For a given window-size *N*, there are (*L - N*) instead of  windows, but these (L-N) windows are not independent from each other. The "effective" number of windows that need to be corrected for multiple tests should fall between (*L - N*) and . The use of (*L - N*) will present an upper bound of the probability *P*. Both *P *-values based on the term  and *L - N *are presented in the output.

Unlike in the RDP program, the size of the sliding window is not fixed in the OrgConv package. Instead, from the site where windows begin, the final window size is from the window that has the smallest *P*-value. This is computationally more expensive than the calculation using a fixed window-size in the RDP program. This computationally expensive calculation is used in the program because there is no easy way for users to pre-set any window-size that will be guaranteed to be optimal for their data. Finally, the performance of the improved calculations and the original RDP method was evaluated via simulation.

### The OrgConv package

**Figure 1:**
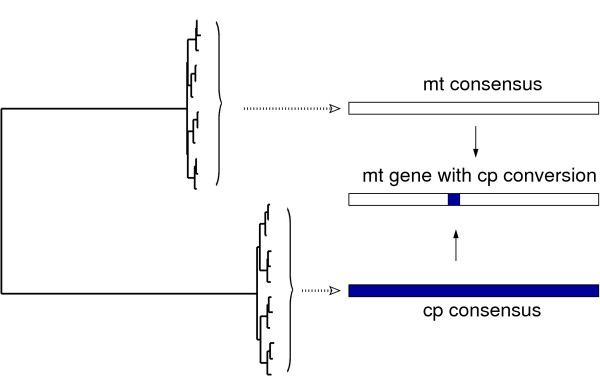
**Detecting gene conversion between plant mitochondrial and chloroplast homologous genes**. In a more general context, gene conversion may also be detected using consensus sequences when the within-clade divergence is low and between-clade divergence is high.

The OrgConv package contains five programs, mtcpconv, twopop, onepop, seq3comp, and seqconsen (see Additional file 1). The C++ source code is available at the OrgConv website and can be installed and run on a Linux platform. Input files for mtcpconv, twopop are two multiple sequence alignments from two examined groups in FASTA format, while input files for the other three programs are single multiple sequence alignment in FASTA format (see Additional file 1). The mtcpconv program first constructs a consensus sequence for each sequence alignment (e.g. mt and cp) and then compares every examined sequence against the two consensus sequences (illustrated in Figure 1). Comparing examined sequences against the consensus sequences rather than examining every possible combination of three sequences greatly reduces computational burden and it gives mtcpconv a great advantage over other programs of analyzing large data sets because of computational time savings. The rationale of using consensus sequences is that plant mitochondrial genes have extremely low nucleotide substitution rates [22,23], so regions transferred from chloroplast into mitochondria should be notably different from other mitochondrial genes but highly similar with chloroplast genes. Nonetheless, the use of consensus sequences, while computationally advantageous, increases the chance that recombination events involving chloroplast regions that differ significantly from the chloroplast consensus sequence will be missed (as opposed to comparisons with the actual donor sequence or sequences closely related to it). To account for this possibility, users have the option to examine every possible sequence combination using the twopop or onepop program. The twopop program was developed to search for gene conversion between two distinct groups, while onepop was developed to detect gene conversion within a single group. The latter works in a way similar to the RDP program [3] (see Additional file 1 for more details). Two additional programs, seq3comp and seqconsen, are also included in the OrgConv package: These allow detection of gene conversion from sequence triplets and construction of consensus sequences from alignments.

### The OrgConv web interface

**Figure 2:**
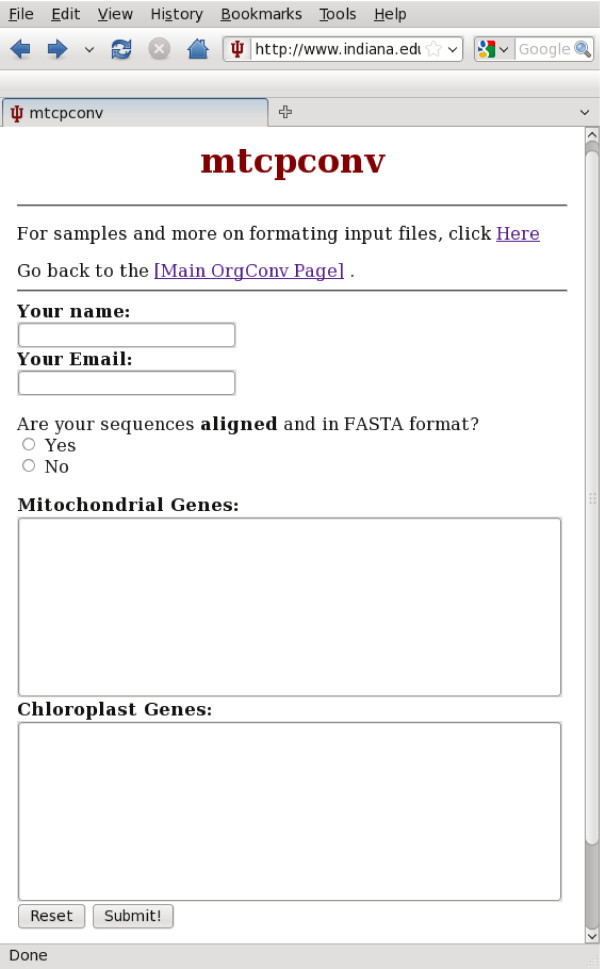
**Web interface where mt and cp alignments could be uploaded**.

**Figure 3:**
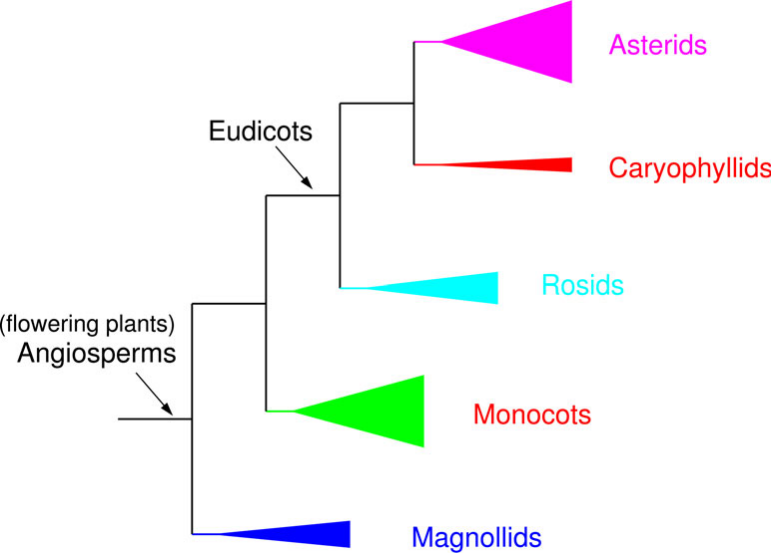
**Five groups of flowering plants examined in the study**. The phylogenetic relationship was derived from refs. 39 and 40. The edge length of each group is proportional to the number of mitochondrial genes listed in Table 2.

**Figure 4:**
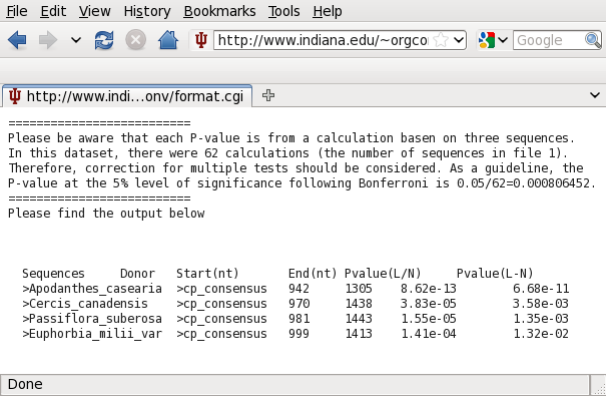
**mtcpconv output for the Rosids group**. As described in the text, the problem of multiple windows was corrected in two ways, one is to use (*L - N*) and the other is to use , both P-values are presented in the output and the segments with both initial *P *-values < 0.05 (before Bonferroni correction) are shown.

A user-friendly web interface that uses Perl CGI scripts is accessible via a variety of web browsers (e.g. Firefox, IE, Safari, and Konqueror) and on multiple operating systems (Windows, Mac, and Linux). The web interface currently runs mtcpconv, seq3comp, and seqconsen. To run programs on the web interface, users just need to upload sequence alignments, and results are displayed in HTML format when the analysis is finished. A snapshot of the mtcpconv interface is shown in Figure 2. Analysis was conducted using the mitochondrial *atp1 *and chloroplast *atpA *data from ref. [28]. The output for the Rosids group (a group of flowering plants, Figure 3) is shown as an example in Figure 4. Although written in Perl CGI scripts, the web-interface calls executables compiled from C++ source codes for the main computations. Thus, the computational performance on the web-server should be comparable to installing and running the distributed source code on a local computer.

## Results and Discussion

**Figure 5:**
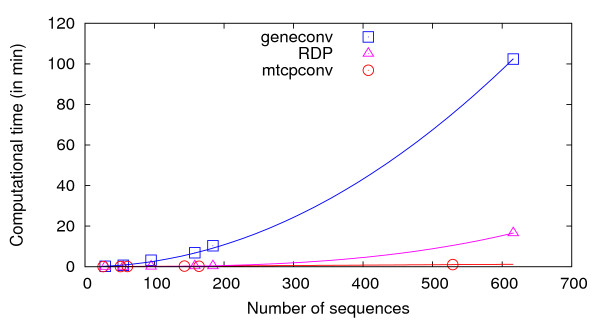
**Association between computational time and the number of sequences using different programs**. Data are from Table 2. When using geneconv and RDP, mt and cp sequences were combined, thus the number of sequences shown for geneconv and RDP is the number of both mt and cp sequences. Regression line was fit using each set of data with a restriction of passing through the origin (0,0 point).

**Figure 6:**
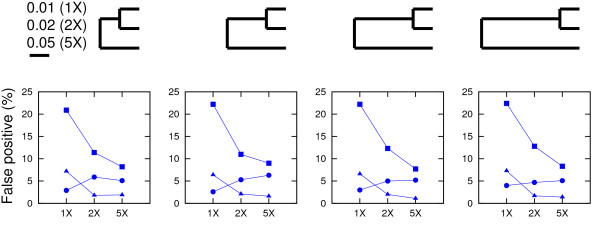
**Rate of false positives using different calculations when no recombination was introduced**. Calculations strictly following the RDP equation are shown in circles, improved calculations using  to correct for multiple windows are shown in squares and improved calculations using (*L - N*) to correct for multiple windows are shown in triangles. Each data point is the average from 1000 iterations.

**Figure 7:**
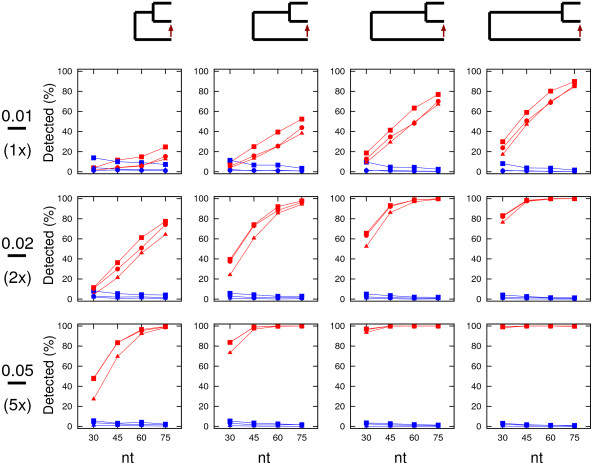
**Power (red) and rate of false positives (blue) using different calculations when one recombination was introduced**. A recent recombination was introduced on each phylogeny in four different sizes (30 nt, 45 nt, 60 nt, and 75 nt). Symbols for the three calculations are as in Figure 6. Each data point is the average from 1000 iterations.

**Figure 8:**
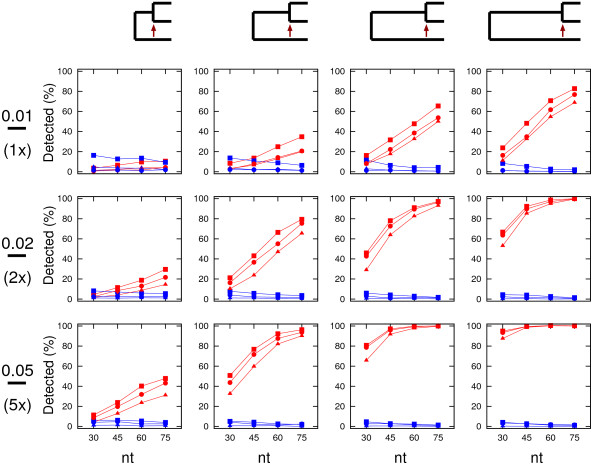
**Power (red) and rate of false positives (blue) using different calculations when one recombination was introduced**. A relatively ancient recombination was introduced on each phylogeny in four different sizes (30 nt, 45 nt, 60 nt, and 75 nt). Symbols for the three calculations are as in Figure 6. Each data point is the average from 1000 iterations.

Unlike RDP that can only be run on a Windows operating system, OrgConv has the advantage that it can be installed and run on a Linux platform (including the Linux environment on Mac) and the web interface is accessible on multiple operating systems (Windows, Mac, and Linux). Besides the different operating systems the programs run on, a main difference between mtcpconv and RDP is that mtcpconv makes use of consensus sequences. In principle, the same goal can be achieved using RDP. First, consensus sequences need to be generated from the two alignments, then the calculation for the *P*-value can be conducted on the two consensus sequences and one examined sequence each time using RDP. Since mtcpconv and RDP use the same method to calculate the probability, the results using the two programs should be very similar if not identical. However, the current version of RDP does not have the option to compare consensus sequences automatically. In practice, it would be difficult for users to conduct such analyses on a large number of sequences only using RDP. As the number of sequences in a given analysis increases, the total number of possible sequence combinations increases exponentially (Table 1). Comparing every examined sequence against the consensus sequences as done in mtcpconv instead of examining every possible sequence combinations greatly reduces the number of comparisons. The computational performance of mtcpconv was evaluated and compared with other programs geneconv and RDP using *atp1 *- *atpA *homologs in different groups of flowering plants [28]. As the number of sequences increases, the computational time increases linearly with mtcpconv instead of quadratically using geneconv and cubically using RDP (Table 2 and Figure 5). For example, on a 3 GHz Intel vPro, 3 GB RAM, Linux Fedora 10 machine, mtcpconv requires about 1 min to analyze a 529 mitochondrial *atp1 *alignment and an 87 chloroplast *atpA *alignment (1,629 characters in length). On the same data, the computational time is 102 minutes 21 seconds using geneconv and 16 minutes 33 seconds using RDP. RDP is a Windows program and it was run on a 2.2 GHz AMD Athlon, 2 GB RAM, Windows XP machine. Even though RDP was run on a different machine and on a different operating system, the substantial difference in computational time (Figure 5) is sufficient to suggest that mtcpconv outperforms both geneconv and RDP when the number of sequences is large. More importantly, the reduction of comparisons using consensus sequences provides the added benefit of greatly increasing statistical power. Generally, when the number of calculations is large, there is a multiple testing problem because of the increased risk of getting false positives by chance. The multiple testing problem is usually treated by some correction method, such as the widely used Bonferroni correction. For instance, when there are *k *comparisons, the observed *P*-value considered to be significant at the 0.05 level is  after Bonferroni correction. When the number of calculations is large, the initial *P*-value needs to be extremely small to be considered significant (Table 1). On the *atp1 *- *atpA *data, after Bonferroni correction, most of the mt-cp conversion segments detected by mtcpconv are not significant using geneconv and RDP (Table 2). To obtain a conservative view of the performance of mtcpconv, both *P*-values are required to be significant. To illustrate the statistical power of mtcpconv on large datasets, I compared its performance to RDP and geneconv with the entire angiosperms (flowering plant) *atp1 *- *atpA *data and a smaller subset of the data using just the asterids group (Figure 3). The phylogenetic relationship of asterids and other four groups of angiosperms was derived from [29,30] (Figure 3). When applied to the same data in the Asterids group, mtcpconv detected 28 recombinant segments, geneconv detected 17 recombinant segments, and RDP did not detect any segments (Table 2). When applied to a larger dataset (all flowering plants), geneconv did not detect any segments, and even the ones previously detected in the Asterids group became non-significant. In contrast, mtcpconv detected 34 segments in all angiosperms and 27 out of the 28 previously detected segments in the Asterids group remain significant. RDP only detected one segment, which is in the flowering plant *Apodanthes casearia *(shown as the most significant one in Figure 4). It was significant in both the Rosids group and all angiosperms. These results suggest that mtcpconv is powerful on large datasets in the face of Bonferroni correction. Given the lack of sensitivity of existing programs, it is not surprising that mt-cp conversion events were previously overlooked. The performance of the core calculation used in the RDP program and the improved ones used in this study was evaluated via simulations. In brief, simulation was conducted on a series of three-taxon phylogenies with four different branch length ratios and three different levels of divergence for each branch length ratio. Sequences were simulated to have 1500 nt in length on the basis of each phylogeny using the Seq-Gen program [31] with equal base composition and the transition/transversion ratio equal to 2. First, I evaluated the false positive rates of different calculations (Figure 6). Sequences were generated with no recombination introduced and then scanned for recombination. Any detected recombinant region would be deemed as a false positive. It is clear that the false positive rate is the highest when using the improved *p *and the term  to correct for multiple windows. Furthermore, the false positive rate is negatively associated with the degree of divergence between the two closely related taxa. When their branch length is 0.01 (pairwise distance equal to 0.02), the false positive rate could be up to 22% when the term  was used to correct for multiple windows, and close to 7% when the term *L - N *was used to correct for multiple windows. The high rate of false positives in low diverse sequences could be explained by the high stochastic error in the *p *calculation. For instance, when the degree of divergence is low, the *p *excluding the examined region could be stochastically much smaller than the *p *from the entire sequence. It is important to mention that the detected recombinant regions listed in Table 2 are not likely false positives. 1), calculations using *L - N *to correct for multiple windows have lower false positive rates than those using  to correct for multiple windows. 2), there is a reasonable degree of divergence within each group. For the groups that have recombinant segments, the average DNA distance between the consensus and each sequence is 0.023 in Rosids, 0.031 in Asterids, and 0.047 in all angiosperms. 3), the P-values are remarkably significant for most segments. For instance, in all angiosperms, 34 segments are significant at the 0.05 level after Bonferroni correction. 33 of them are significant at the 0.01 level, and 30 of them are significant at the 0.001 level. Second, I evaluated the power of different calculations (Figures 7 and 8). Sequences were simulated with one recombination introduced at the beginning of the sequence. If a putative recombinant region inferred from the calculation that is within the simulated recombinant region was deemed as a correct detection, otherwise it was deemed as a false positive. It is clear that the power of recombination detection increases as the degree of divergence increases and the power increases as the distant sequence is more diverse from the other two sequences. Furthermore, the power is higher when the recombination is recent compared with when the recombination is ancient.

**Table 1:** Substantially increased comparisons and decreased *P*-values to be considered significant for Bonferroni correction along with the increase of sequences if every possible combination is calculated

**Using 2 sequences (e.g. ****geneconv**)			**Using 3 sequences (e.g. **RDP)		
combination	number(k)	0.05/*k*	combination	number(k)	0.05/*k*
	5.0 × 10^3^	1.0 × 10^-05^		1.6 × 10^5^	3.1 × 10^-07^
	5.0 × 10^5^	1.0 × 10^-07^		1.7 × 10^8^	2.9 × 10^-10^
	5.0 × 10^7^	1.0 × 10^-09^		1.7 × 10^11^	2.9 × 10^-13^

**Table 2:** Performance of mtcpconv, geneconv, and RDP on a various number of mitochondrial atp1 and chloroplast atpA sequences in different angiosperm groups

	sequences		Computational time^†§^			mt-cp segments detected		
Sequence group^‡^	mt	cp	**mtcpconv**	**geneconv**	**RDP**	**mtcpconv**	**geneconv**	**RDP**
Caryophyllids	26	3	1.42s	9.70s	0.50s	0	0	0
Magnoliids	51	4	2.11s	39.24s	0.36s	0	0	0
Rosids	61	34	4.62s	3m9s	3.95s	1	0	1
Monocots	143	15	17.72s	6m52s	17.16s	0	0	0
Asterids	164	20	13.62s	10m18s	17.84s	28	17	0
All angiosperms	529	87	1m4.12s	102m21s	16m33s	34	0	1

^†^mtcpconv and geneconv were run on a 3 GHz Intel vPro, 3 GB RAM, Linux Fedora 10 machine, while RDP was run on a 2.2 GHz AMD Athlon, 2 GB RAM, Window XP machine.

^§^mt sequences and cp sequences were combined into one file when analyzed using geneconv and RDP.

^‡^The relationship of the five groups is shown in Figure 3.

The use of consensus sequences carries a risk that recombination events involving chloroplast regions that differ significantly from the chloroplast consensus sequence will be missed. A possible approach to overcoming this is to compare mitochondrial sequences against chloroplast sequences from closely related species. Indeed, 23 segments in the Asterids group did show slightly smaller initial (before Bonferroni correction) *P*-values when comparing Asterids mitochondrial and chloroplast sequences than comparing all angiosperm mitochondrial and chloroplast sequences (data not shown). However, comparison of sequences from the same taxonomic group might not always outperform comparison of larger groups. For example, *Myrtus communis *has been detected to have a mtcp-conversion by analyzing the entire angiosperms dataset with two *P*-values of 6.19 × 10^-08 ^and 4.70 × 10^-06^, whereas the *P*-values when comparing Rosids mitochondrial and chloroplast genes are only 2.74 × 10^-03 ^and 2.50 × 10^-01^, and not considered to be significant in Figure 4.

**Figure 9:**
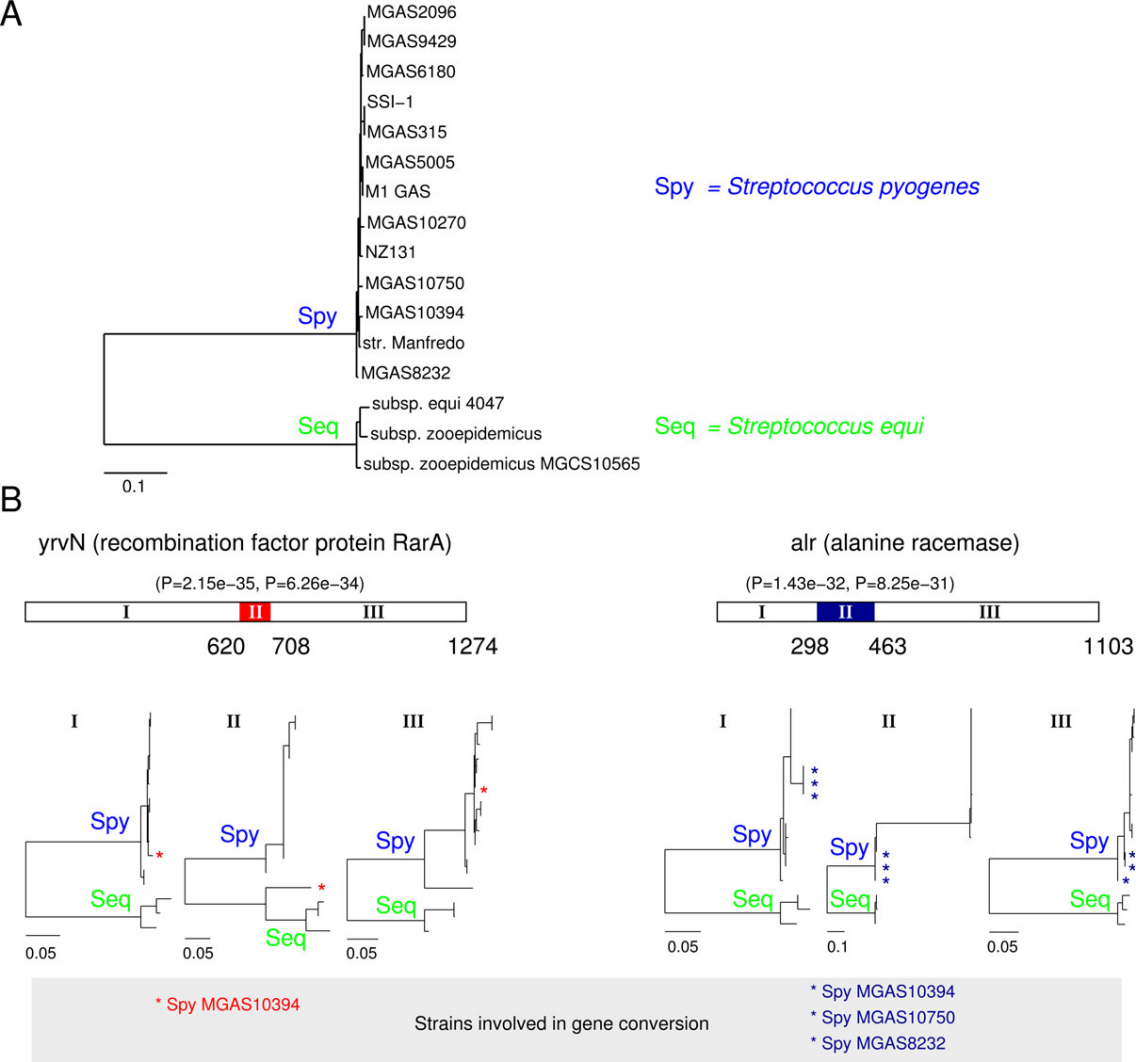
**Application of ****mtcpconv****on two groups of Streptococcus strains**. A, phylogeny of the concatenated sequences of 232 genes (with 249,790 characters). *S. pyogenes *and *S. equi *strains form two distinct groups with little diversity within each group; B, two most significant genes involved in gene conversion detected by the mtcpconv program.

Even though the mtcpconv program was developed with main focus on detection of mt-cp conversion, its application can extend beyond mitochondrial and chloroplast genes. The prerequisite of the program is the use of consensus sequences and it relies on the fact of low diversity within both mitochondrial genes and chloroplast genes but much diversity between them. Ideally, if two groups of sequences have very little diversity within each group and much diversity between groups, mtcpconv would be suitable to use for detection of conversion between the two groups. Knowing the existence of recombination in *Streptococcus *strains [32-34], I applied mtcpconv to 232 commonly present genes (my unpublished data) to detect gene conversion between two *Streptococcus *species *pyogenes *and *equi *(Figure 9). Two genes with the smallest *P*-values from the mtcpconv program are shown in Figure 9B, and further phylogenetic analysis supports the chimeric nature of these genes. In conclusion, mtcpconv seems to work well when two groups of sequences have little diversity within each group and much diversity between groups. However, when there is little diversity between groups or very much diversity within each group, mtcpconv is not expected to work well. In such a case, programs for more general recombination/conversion detection, such as RDP, geneconv, and Max*χ*^2^, should be considered. Furthermore, mtcpconv along with other programs in the OrgConv package assumes that the substitution rate of a nucleotide position is constant throughout time (i.e., in all lineages), although the rate can vary between positions. In deed, however, the substitution rate of sites in a gene can change over time, this is also known as heterotachy [35]. Heterotachy could potentially lead to false positives when using programs in the OrgConv package. Under such circumstances, more explicitly phylogenetic methods such as LIKEWIND[15], TOPAL[36], and bootscanning [37] are likely to work better.

## Conclusions

The OrgConv package was developed for detection of mt-cp conversion. It makes use of the consensus sequence from each group of sequences and compare each examined sequence against consensus sequences rather than examining every possible sequence combination. By doing so, computational burden has been significantly reduced and it becomes feasible to analyze very large data sets. More importantly, the statistical power of the program is retained in the face of Bonferroni correction because of the reduced number of comparisons. Furthermore, although developed for detection of mt-cp converson, the program may be applied on other sequences than mitochondrial and chloroplast sequences, e.g., when two large groups of sequences have very low diversity within each group and high diversity between groups.

## Availability and requirements

• **Project name: **OrgConv

• **Project home page: **http://www.indiana.edu/~orgconv

• **Operating system(s): **Linux for the distributed source code and operating systems independent for the web-interface

• **Programming language: **C++ for the source code and Perl CGI scripts for the web-interface

• **License: **Free for academic use

## List of abbreviations

HGT: horizontal gene transfer; HR: homologous recombination; cp: chloroplast; mt: mitochondria; mt-cp conversion: gene conversion between mitochondrial and chloroplast genes.

## Authors' contributions

WH designed the study, wrote the programs, and wrote the manuscript.

## Supplementary Material

Additional file 1 **OrgConv****manual**. The manual for OrgConv. Click here for file
