# Supplementary material for: OrgConv: detection of gene conversion using consensus sequences and its application in plant mitochondrial and chloroplast homologs
Source: BMC Bioinformatics. 2010 Mar 2;11:114. doi: 10.1186/1471-2105-11-114 (PMC2842260; doi:10.1186/1471-2105-11-114)
Supplement: Additional file 1 — OrgConvmanual. The manual for OrgConv. [file 1471-2105-11-114-S1.PDF]

OrgConv Manual

Weilong Hao
HaoWeilong@gmail.com

January 13, 2010

1 Overview

OrgConv (Organellar Conversion) is a program designed for detection of gene conversion between plant mitochondrial and chloroplast homologous genes (or mt-cp conversion). Even though it was developed for detection of gene conversion between plant organelles, OrgConv has a good potential to be used more broadly e.g. between any two distinct groups of sequences. The package contains a feature program mtcpconv, and 4 other programs onepop, twopop, seqconsen, and comp3seq. OrgConv is available at the OrgConv website (http://www.-indiana.edu/˜orgconv).

The core calculation for detection of conversion in mtcpconv, onepop, twopop, and comp3seq was conducted using a method modified from the RDP (Recombination Detection Program) method (Martin and Rybicki 2000). In brief, the RDP method compares three sequences each time by only examining informative sites. The probability to observe one recombination follows a binomial distribution:

$$P = \frac{L}{N} \times \sum_{m=M}^{N} \Big(\frac{N!}{m!(N-m)!}\Big) p^m \times (1-p)^{N-m},$$

where L is the length of informative sites, N is the length of the putative recombinant segment, M is the common nucleotides shared between the putative recombinant sequences, p is the proportion of nucleotides common between the same pair of sequences.

In the package, two improvements were made to the above calculation. 1), the parameter p (the proportion of nucleotides common between sequences) was calculated from the sequence excluding the examined region instead of from the entire sequence. The calculation in the original RDP method is under the null hypothesis that there is no recombination. However, when there is recombination, the proportion of nucleotides common between the entire donor and recipient sequences is inflated because of the recombinant region, and consequently the calculated probability P will be less significant than it should be. It would therefore be more appropriate to exclude the examined region from the overall p calculation. 2), in addition to $\frac{L}{N}$, a second term $(L-N)$ was introduced for multiple window correction. In this study, calculation was performed in sliding-windows by incrementing one informative site at a time. For a given window-size N, there are $(L-N)$ instead of $\frac{L}{N}$ windows, but these (L-N) windows are not indepedent from each other. The "effective" number of windows that need to be corrected for multiple tests should fall between $(L-N)$ and $\frac{L}{N}$. The use of $(L-N)$ will present an upper bound of the probability P. Both P-values are presented in the output, one is labeled as P-value($L-N$) and the other is labeled as P-value(L/N).

In the `mtcpconv` program, computational burden is significantly reduced by using the consensus sequence from each population rather than examining every possible combination of three sequences. The rationale of using consensus sequences is that plant mitochondrial genes have extremely low substitution rates (Wolfe *et al.* 1987), regions transferred from chloroplast into mitochondria should be remarkably different from any other mitochondrial genes but highly similar with chloroplast genes (Hao and Palmer 2009). The use of consensus sequences in cases like this would not affect the accuracy of the analysis but rather bring in an advantage due to the greatly reduced number of comparisons/calculations. As the number of sequences increases, the number of possible sequence combination increases exponentially (see calculations below), and then it becomes extremely difficult to examine every possible sequence combination as done generally, such as in `GENECONV` (Sawyer 1989) or `RDP` (Martin and Rybicki 2000).

$$\binom{10}{2} = 4.5\times10^{01} \qquad \binom{10}{3} = 1.2\times10^{02}$$
$$\binom{100}{2} = 5.0\times10^{03} \qquad \binom{100}{3} = 1.6\times10^{05}$$
$$\binom{1000}{2} = 5.0\times10^{05} \qquad \binom{1000}{3} = 1.7\times10^{08}$$
$$\binom{10000}{2} = 5.0\times10^{07} \qquad \binom{10000}{3} = 1.7\times10^{11}$$

Using consensus sequences instead of comparing all possible sequence combinations can greatly reduce the number of comparisons and ultimately reduce the computational burden. For example, on a 3 GHz Intel vPro, 3 GB RAM, Linux Fedora 10 machine, it takes about 1 minutes for a 529 mitochondrial *atp1* alignment and an 87 chloroplast *atpA* alignment (1,629 characters in length). When the number of calculations is large, there is an increased risk of getting false positives by chance. This is usually treated by correction for multiple tests. However, commonly used methods such as Bonferroni correction have been shown to be too conservative when the number of tests gets very large (Nakagawa 2004). Reducing the number of calculations can therefore improve the statistical power. When there are more than 100 sequences, the statistical power can be improved by several orders of magnitude.

Despite the benefits of using consensus sequences, users do have the option of examining every possible sequence combination. `twopop` reads the data in the same way as `mtcpconv` but conducts analysis on every possible sequences combination. Both `mtcpconv` and `twopop` detect gene conversion between two distinct groups of sequences. `onepop` was developed for detection of gene conversion within one group of sequences. `onepop` examines every possible combination within the group, which is in a way similar to the RDP program (Martin and Rybicki 2000). As mentioned above, `onepop` and `twopop` have to conduct a much larger number of calculations and will take longer computational time and the large number of calculations can reduce the statistical power if correction for multiple test such as Bonferroni correction is used. However, `onepop` and `twopop` would be ideal for identifying parent sequences using a reduced number of sequences.

2 Using OrgConv

2.1 Downloading and preparing the executable

Download the source code of `OrgConv` from http://www.indiana.edu/˜orgconv. To use the program please follow the steps below:
1) **download** the source code by clicking the link to the file *orgconv.tar.gz*. Save this file in a separate directory.
2) **unzip** this source code using the following command:

```
tar -zxvf orgconv.tar.gz
```

This should expand the source code in a directory named *OrgConv*. Change directory to the new directory using

```
cd OrgConv
```

3) **make** executable files by typing *make* while in the *OrgConv* directory.

```
make
```

This will result in an executable file called `mtcpconv`. If you think you might be using other programs (`onepop`, `twopop`, `seqconsen`, `comp3seq`) as well, please type:

```
make onepop
make twopop
make seqconsen
make comp3seq
```

Then type:

```
make clean
```

2.2 Running the executable

Programs mentioned above might require different number of data files and they are described below:

2.2.1 mtcpconv

`mtcpconv` reads two files, one contains mitochondrial genes and the other contains chloroplast genes (or genes from two distinct populations). To run the program, type:

./mtcpconv <data-file-1> <data-file-2>

<data-file-1> and <data-file-2> (‘<’ and ‘>’ are not part of the command) are the two files you are going to analyze. Please note that the program does not make corrections for multiple tests during the calculation. Upon finish, it will print a guideline message (see the ‘Output files’ section) as standard output following the Bonferroni correction. You can redirect the standard output into a file <outfile> by typing:

./mtcpconv <data-file-1> <data-file-2> > <outfile>

Putative recombinant regions (when $P < 0.05$) are in a file called *recomb.output*. For the Bonferroni correction, please refer to the guideline message.

`mtcpconv` and `twopop` (described below) only detect gene conversion from <data-file-2> to <data-file-1>. If you are interested in examining gene conversion in both directions, you might need to run the programs one more time by reversing the two files in order:

./mtcpconv <data-file-2> <data-file-1> > <outfile>

2.2.2 twopop

`twopop` reads two files, one contains mitochondrial genes and the other contains chloroplast genes (or genes from two distinct populations). To run the program, type:

./twopop <data-file-1> <data-file-2> > <outfile>

As mentioned above, the <outfile> will contain a guideline message for multiple tests following the Bonferroni correction. Putative recombinant regions (before the Bonferroni correction) are in a file called *recomb.output*.

2.2.3 onepop

`onepop` reads only one file. To run the program, type:

./onepop <data-file-1> > <outfile>

The <outfile> will contain a guideline message for multiple tests following the Bonferroni correction. Putative recombinant regions (before the Bonferroni correction) are in a file called *recomb.output*.

2.2.4 seqconsen

`seqconsen` reads one file and generate a consensus sequence based on the file. To run the program, type:

./seqconsen <data-file-1> > <outfile>

2.2.5 comp3seq

`comp3seq` reads one file that contains 3 sequences and calculates probability for putative recombinant regions. To run the program, type:

./comp3seq <data-file-1> > <outfile>

2.3 Input files

All programs can only read **aligned** sequences in FASTA format. For example,

```
>1seq
--ATATCGGACCTTTGCGACATTTCCCAAATTT
>2seq
ACCTTTGGGACCTTTGGGACCTTTG--ACCTTT
>3seq
AAATTTCCCAAATTACCCTAATTTCCCAAATTT.
```

Sequence names that are larger than 20 characters will be truncated to 20 characters in the output. For mtcpconv and `twopop`, corresponding sites in the two input files are required to be homologous sites, thus the sequences in the two input files should be generated from one set of alignments.

2.4 Output files

The standard output of `mtcpconv`, `onepop`, `twopop` looks something like below with a guideline for multiple tests adjustment.

```
==========================
Please be aware that each P-value is from a calculation based on three sequences.
In this dataset, there were 13 calculations (the number of sequences in mt.fasta).
Therefore, correction for multiple tests should be considered.  As a guideline, the
P-value at the 5% level of significance following Bonferroni is 0.05/13=0.00384615.
==========================
The output is in a file called recomb.output!
```

Putative conversion regions are written in a file called *recomb.output*. The file *recomb.output* looks something like this

Sequences	Donor	Start(nt)	End(nt)	Pvalue(L/N)	Pvalue(L-N)
>Apodanthes_casearia	>cp_consensus	942	1305	8.62e-13	6.68e-11
>Myrtus_communis	>cp_consensus	970	1438	3.83e-05	3.58e-03
>Euphorbia_milii	>cp_consensus	999	1413	1.41e-04	1.32e-02
>Passiflora_suberosa	>cp_consensus	981	1443	1.55e-05	1.35e-03

`comp3seq` will print out putative conversion regions as standard output like above. `seqconsen` will print out the consensus sequence as standard output as well.

References

Hao W, Palmer JD. 2009. Fine-scale mergers of chloroplast and mitochondrial genes create functional, transcompartmentally chimeric mitochondrial genes. Proc Natl Acad Sci U S A 106:16728–16733.

Martin D, Rybicki E. 2000. RDP: detection of recombination amongst aligned sequences. Bioinformatics 16:562–563.

Nakagawa S. 2004. A farewell to Bonferroni: the problems of low statistical power and publication bias. Behav Ecol. 15(6)1044–1045.

Sawyer S. 1989. Statistical tests for detecting gene conversion. Mol Biol Evol 6:526–538.

Wolfe KH, Li WH, Sharp PM. 1987. Rates of nucleotide substitution vary greatly among plant mitochondrial, chloroplast, and nuclear DNAs. Proc Natl Acad Sci U S A 84:9054–9058.
